# EZH2 identifies the precursors of human natural killer cells with trained immunity

**DOI:** 10.20892/j.issn.2095-3941.2020.0791

**Published:** 2021-09-24

**Authors:** Chen Zhang, Jie Yin, Jian Zheng, Jun Xiao, Jiajian Hu, Yudong Su, Kaichen Zhou, Yingchi Zhang, Xuzhen Zhang, Hong Zhang, Qian Sun, Yang Wang, Wenwen Yu, Feng Wei, Qiang Zhao, Long Li, Xiubao Ren

**Affiliations:** 1 Department of Immunology and Biotherapy, Key Laboratory of Cancer Immunology and Biotherapy, Tianjin Medical University Cancer Institute and Hospital, National Clinical Research Center for Cancer, Key Laboratory of Cancer Prevention and Therapy, Tianjin, Tianjin’s Clinical Research Center for Cancer, Tianjin 300060, China; 2 Department of Immunology, Key Laboratory of Immune Microenvironment and Disease of the Ministry of Education, Tianjin Medical University, Tianjin 300070, China; 3 Department of Pediatric Cancer, Tianjin Medical University Cancer Institute and Hospital, Tianjin 300060, China; 4 State Key Laboratory of Experimental Hematology and Division of Pediatric Blood Diseases Center, Institute of Hematology and Blood Diseases Hospital, Chinese Academy of Medical Sciences and Peking Union Medical College, Tianjin 300000, China; 5 Center for Stem Cell Medicine, Chinese Academy of Medical Sciences, Beijing 100730, China; 6 Research Center of Basic Medicine Science, Tianjin Medical University, Tianjin 300070, China

**Keywords:** Natural killer cells, trained immunity, precursor, EZH2, cell cycle

## Abstract

**Objective:**

Trained immunity of natural killer (NK) cells has shown great potential in the treatment of cancers by eliciting enhanced effector responses to restimulation by cytokines or cancer cells for long time periods after preactivation. However, the human NK cells responsible for the generation and maintenance of trained immunity are largely unknown. We hypothesized that heterogeneous human NK cells would respond differentially to stimulation with a combination of IL-12, IL-15, and IL-18, and that an NK cell subset might exist that is mainly responsible for the induction of trained immunity. On the basis of our hypothesis, we aimed to identify the subset from which cytokine-trained human NK cells originate and to explore possible regulatory targets for drug intervention.

**Methods:**

Flow cytometry assays were performed to analyze the functions of cytokine-trained NK cells and examine cell division and protein expression in NK cell subsets. Single-cell RNA sequencing (scRNA-seq) plus TotalSeq™ technology was used to track the heterogeneity of NK cells during the induction of trained immunity.

**Results:**

Traditional developmental markers for peripheral NK cells were unable to identify the precursors of human NK cells with trained immunity. Therefore, we used scRNA-seq plus TotalSeq™ technology to track the heterogeneity of NK cells during the induction of trained immunity and identified a unique cluster of CD57^−^NKG2A^+^EZH2^+^IFNG^+^MKI67^+^IL12R^+^IL15R^+^IL18R^+^ NK cells. Enrichment and pseudotime trajectory analyses suggested that this cluster of NK cells contained the precursor of trained NK cells. We then used flow cytometry to further investigate the role of EZH2 in trained NK precursors and found that CD57^−^NKG2A^+^EZH2^+^ NK cells had faster cell cycles and an enhanced trained phenotype, and EZH2 inhibition significantly impaired the induction of trained immunity in NK cells. These results suggested that EZH2 is a unique epigenetic marker of precursors of human NK cells with trained immunity.

**Conclusions:**

Our work revealed human NK heterogeneity in the induction of trained immunity, identified the precursor subset for trained NK cells, and demonstrated the critical role of EZH2 in the induction of trained immunity in human NK cells.

## Introduction

Natural killer (NK) cells are cytotoxic innate lymphoid cells that are important in the host defense against pathogens and antitumor immune responses^1,2^. Adoptive transfer of NK cells into cancer patients is a major approach in NK cell-based therapy; however, this therapy requires further improvement^3,4^. The cytokines IL-2, IL-12, IL-15, IL-18, IL-21, and type I interferons have been used to expand and activate NK cells *in vitro* before adoptive transfer, and this process efficiently boosts the quantity and function of NK cells^3^. Among these cytokines, the combination of IL-12, IL-15 and IL-18 (IL-12/15/18) is particularly effective because of its ability to induce trained immunity of NK cells. This immunity has potent effector functions and shows long-term persistence in mice^5^ and humans^6^.

Trained immunity, as recently proposed by Netea et al.^7^, expands immunological memory from adaptive to innate immunity, as supported by a considerable amount of evidence. Innate immune cells with trained immunity have enhanced effector responses when restimulated. A unique property of trained immunity is antigen nonspecificity, because secondary stimuli that trigger enhanced responses need not be associated with the primary stimuli, thus underscoring the antitumor therapeutic potential of the cytokine-induced trained immunity of NK cells. Cytokine-induced memory-like NK cells were first reported in mice. Short-term activation of splenic NK cells with IL-12 and IL-18 (IL-12/18) gives rise to a population of cells with durable enhanced production of IFN-γ in response to restimulation with cytokines, thereby activating receptor ligands or tumor targets^5^. This example is typical of trained immunity in NK cells. Human NK cells also have a similar ability to generate cytokine-induced trained immunity^6^. Human NK cells preactivated with IL-12/15/18 show enhanced IFN-γ production in response to restimulation with cytokines or tumor target cell lines^6^. Cytokine-trained human NK cells xenografted into mice substantially decrease AML burden *in vivo* and improve overall survival^8^. In addition, a previous study has reported enhanced IFN-γ production and cytotoxicity of cytokine-trained human NK cells against ovarian cancer cells *in vitro* and in a xenogeneic mouse model^9^. Thus, the cytokine-trained immunity of NK cells is emerging as a promising immunotherapy approach for patients with cancer.

The phenotypes of cytokine-trained NK cells are fairly clear. Both the CD56bright and CD56dim NK cell subsets exhibit trained immunity, and the enhanced IFN-γ response is associated with the expression of CD94, NKG2A, NKp46, and CD69, as well as a lack of KIRs and CD57^6^. Further study has confirmed the increased expression of CD25, NKp30, NKp44, CD62L, CD27, TRAIL, perforin, and granzyme B, and the decreased expression of NKp80, in trained NK cells^8^. This list of markers is highly similar to that for naïve human NK cells. Thus, the precursor NK cells that develop into cytokine-trained human NK cells must be differentiated from “naïve” NK cells after exposure to primary stimuli. However, both groups of NK cells share many common markers. In this study, we sought to identify the precursors of trained human NK cells in a heterogeneous NK population that received only primary stimuli. By combining single-cell RNA sequencing (scRNA-seq) with TotalSeq™ for 10 cell-surface antigens and multicolor flow cytometry, we investigated NK cell heterogeneity during cytokine induction of trained immunity. We found that a unique CD57^−^ NKG2A^+^EZH2^+^IFNG^+^MKI67^+^IL12R^+^IL15R^+^IL18R^+^ NK subset was the major source of cytokine-trained NK cells. Our previous work has revealed that EZH2 regulates the differentiation and function of NK cells, and its expression levels correlate well with NK cell development^10^. Here, we demonstrate that EZH2 is also involved in the induction of cytokine-trained NK cells. Our findings indicate the importance of the CD57^−^ NKG2A^+^EZH2^+^IFNG^+^MKI67^+^IL12R^+^IL15R^+^IL18R^+^ NK subset in the development of trained immunity of NK cells. This subset may therefore have potential as a drug target in clinical applications.

## Materials and methods

### Blood collection and ethics statement

All fresh human blood samples for flow cytometry experiments and scRNA-seq were obtained from the Tianjin Blood Center. For scRNA-seq and TotalSeq™, PBMCs were collected from 3 healthy donors. Only 1 donor sample (human sample 2) passed the quality control analysis on day 7, largely because the 7-day *in vitro* culture made the NK cells too fragile to sustain the standard single-cell capture procedure. All samples were provided anonymously after informed consent was obtained from participants. The collection, distribution, and use of all identified human peripheral blood samples were approved by the Tianjin Medical University Cancer Institute and Hospital (Approval No. Ek2018086).

### Reagents

Flow cytometry antibodies anti-CD56 (HCD56, 318318), anti-CD3 (UCHT1, 300447), anti-CD57 (HCD57, 322315), anti-IFN-γ (B27, 506529), anti-Ki67 (Ki-67, 350519), anti-TNF-α (Mab11, 502915), and anti-Granzyme B (QA16A02, 372207) were from BioLegend (San Diego, CA, USA); anti-NKG2C (REA205, 130-119-776) and anti-NKG2A (REA110, 130-113-563) were from Miltenyi Biotec (Bergisch Gladbach, Germany); and anti-EZH2 (11/EZH2, 562478) was from BD Pharmingen (San Jose, CA, USA). The TotalSeq™ antibodies TotalSeq™-B0034 anti-human CD3 (300477), TotalSeq™-B0063 anti-human CD45RA (304161), TotalSeq™-B0083 anti-human CD16 (302063), TotalSeq™-B0084 anti-human CD56 (NCAM) recombinant (392423), TotalSeq™-B0101 anti-human CD335 (NKp46) (331939), TotalSeq™-B0147 anti-human CD62L (304849), TotalSeq™-B0154 anti-human CD27 (302851), TotalSeq™-B0161 anti-human CD11b (301357), TotalSeq™-B0390 anti-human CD127 (IL-7Rα (351354), and TotalSeq™-B0087 anti-human CD45RO (304257) were obtained from BioLegend (San Diego, CA, USA). The True-Nuclear Transcription Factor Buffer Set (424401) and CFSE Cell Division Tracker Kit (423801) were purchased from BioLegend (San Diego, CA, USA). The CellTrace Violet Cell Proliferation Kit (C34557) was purchased from Thermo Fisher Scientific (Waltham, MA, USA). The EZH2 inhibitor UNC1999 (S7165) was purchased from Selleckchem.com (Houston, TX, USA). The following endotoxin-free recombinant human (rh) cytokines were used: rhIL-12 (PeproTech), rhIL-18 (MBL International), and rhIL-15 (PeproTech). PMA/ionomycin (cell activation cocktail with brefeldin A) was purchased from BioLegend (San Diego, CA, USA).

### Cell culture and NK cell purification

PBMCs from human platelet apheresis donors were isolated from blood samples through Ficoll centrifugation. Human PBMCs were plated at 3–5 × 10^6^ cells/mL and preactivated for 16 h under rhIL-12 (10 ng/mL)+rhIL-18 (50 ng/mL)+rhIL-15 (1 ng/mL) or control (rhIL-15, 1 ng/mL) conditions, washed 3 times to remove cytokines, and cultured in complete RPMI 1,640 medium containing 10% fetal bovine serum supplemented with rhIL-15 (1 ng/mL) to support survival. Half of the medium was replaced every 2–3 days with medium containing fresh cytokines. When purified NK cells were used for scRNA-seq and TotalSeq™, fresh human blood was collected, erythrocytes were removed with hydroxyethyl starch precipitation, and NK cells were enriched with an NK Cell Isolation Kit (130-092-657; Miltenyi Biotec).

### Assays for expression, function, and proliferation

At the indicated time points, cells were harvested and restimulated with IL-12/15/18 or PMA+ionomycin (containing brefeldin A) for 5 h. Cells were then stained for surface NK cell markers and intracellular IFN-γ, TNF-α, GzmB, Ki67, or EZH2, and subjected to flow cytometry analysis. For the proliferation experiments, NK cells were first labeled with CFSE (1 μM; Biolegend) or CellTrace Violet (1 μM; Thermo Fisher Scientific) for 5 min, then cultured, stimulated, stained, and subjected to flow cytometry analysis as indicated.

### Cytotoxicity assays

Enriched human NK cells were preactivated or cultured under control conditions for 16 h, washed 3 times, and then cultured in complete RPMI 1640 medium containing 10% fetal bovine serum supplemented with a low dose of rhIL-15 for 7 days. Cytotoxicity assays were performed by coincubation of cytokine-trained or control NK cells with K562 cells for 4 h and quantitative measurement of lactate dehydrogenase release with a CytoTox 96 Non-Radioactive Cytotoxicity Assay Kit (G1780, Promega).

### Flow cytometric analysis

Cell staining was performed in U-bottom 96-well plates. Briefly, cells were transferred to 96-well plates at approximately 1 × 10^6^/well, incubated with human Fc receptor blocking solution (422302; Biolegend), and then stained with cell viability dye (423106; Biolegend), surface antibodies, and intracellular antibodies. Data were acquired on an LSRFortessa flow cytometer (BD Biosciences) and analyzed in FlowJo v0.6.1 (Tree Star) software. Plots were produced in GraphPad Prism v8.3.0 software.

### scRNA-seq

Enriched NK cells were first incubated with TotalSeq™ antibodies for 30 min, washed 3 times with ice-cold PBS containing 1% bovine serum albumin, and counted with a LUNA-FL™ Dual Fluorescence Cell Counter (Logos Biosystems). Then the cells were loaded on a Chromium Single Cell Controller (10x Genomics) to generate single-cell Gel Bead-In-Emulsions (GEM) by using a Single Cell 3′ Library and Gel Bead Kit v3.1 (10x Genomics, San Francisco, CA, USA) and subjected to library preparation according to the instructions in the Chromium Single Cell 3′ Reagent Kits User Guide (v3.1 Chemistry) with Feature Barcoding technology for cell surface proteins. The libraries were quantified with an Agilent 2100 High Sensitivity DNA kit (Agilent, California, U.S.) and sequenced *via* the Illumina NovaSeq6000 platform with 150 bp paired end reads (PE150) (Illumina, San Diego, CA, USA).

### Data analysis

After sequencing, the raw data from each sample were demultiplexed and aligned to the GRCh38 reference genome, and unique molecular identifier counts were quantified with the 10x Genomics Cell Ranger pipeline (v3.1.0, 10x Genomics). We continued the data analysis with the filtered barcode matrix files by using the Seurat package (v3.2.0). Cells with more than 200 detected genes and less than 10% mitochondrial reads were considered valid cells and retained for downstream analysis. To prevent clusters from being biased by mitochondrial transcript content, we scaled the gene expression values according to the cell mitochondrial transcript content. Next, “LogNormalize” and “mean.var.plot” were applied to normalize and find variable features within the single-cell gene expression data. Clustering and differential expression analysis were performed in the R package Seurat with default parameters. On the basis of the PCElbowPlot, we selected a certain number of principal components for the clustering analysis when the number reached the baseline of the standard deviation of the principal components. We generally used the cluster resolution at which a higher resolution did not result in linear increases in clusters. Cell clusters were visualized with *t*-distributed stochastic neighbor embedding (*t-*SNE). For differential gene expression, we performed a model-based analysis of single-cell transcriptomics (MAST) test74 (log fc ≥ 0.25, min.pct = 0.1, min.diff.pct > 0.15) and selected only the genes with an adjusted *P* value < 0.05. For the pathway enrichment analysis, we used the “enrichGO” function in clusterProfiler (v3.14.3) with gene sets from the Gene Ontology knowledgebase. To predict cellular differentiation, we ordered cells in pseudotime by using Monocle2 (v2.14.0) with default parameters. The “GM_state” function in the Monocle2 package was used to find “root cells” in Cluster 6. All analyses were performed in the R 3.6.1 environment.

### Statistical analysis

Data are shown as the means ± SEM in all graphs, and significant differences were calculated with Student’s *t* test or as indicated. *P* < 0.05 was considered significant. All statistical analyses and plots were produced in GraphPad Prism.

## Results

### NK cells with trained immunity exhibit enhanced effector responses

**Figure 1:**
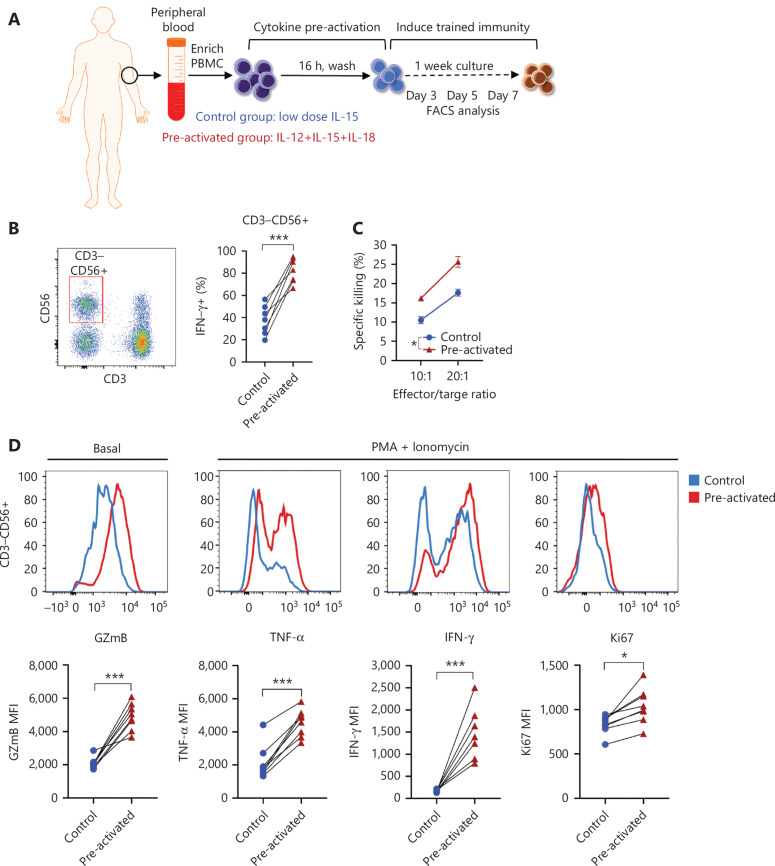
NK cells with trained immunity exhibit enhanced effector responses. (A) Overview of the experimental design. PBMCs were preactivated with IL-12 (10 ng/mL), IL-15 (1 ng/mL), and IL-18 (50 ng/mL), or control (1 ng/mL of IL-15 alone) for 16 h, washed, and then cultured under low concentrations of IL-15 (1 ng/mL) for differentiation. Flow cytometry analysis was performed at the indicated time points after preactivation. (B) Left panel: flow cytometry plot showing the gating strategy for CD3^−^CD56^+^ NK cells; right panel: histogram showing enhanced IFN-γ production by cytokine-trained NK cells restimulated with IL-12/18 on day 7. (C) Increased killing of K562 leukemia target cells by cytokine-trained NK cells on day 7. (D) Upper panel: representative flow cytometry data showing increased basal levels of granzyme B (GzmB) and expression of TNF-α, IFN-γ and Ki67 in cytokine-trained NK cells restimulated on day 7 by PMA+ionomycin; lower panel: summary data showing the median fluorescence intensity (MFI) of GzmB, TNF-α, IFN-γ, and Ki67. For all experiments, *n* = 7, *P < 0.05, **P < 0.01, and ***P < 0.001 (error bars, mean ± SEM). Data are representative of at least 3 independent experiments.

Human NK cells were preactivated with low-dose IL-15 alone as a control or with IL-12/15/18 for 16 h, and then both the control and experimental samples were cultured with IL-15 for 7 more days. The cells were analyzed at different time points, as indicated (**Figure 1A**). As expected, trained CD3^−^CD56^+^ NK cells exhibited enhanced IFN-γ production in response to restimulation for 6 h with IL-12/15/18 on day 7 (**Figure 1B**) and exhibited more efficient killing toward K562 leukemia cells than was observed in the control group (**Figure 1C**). In addition, granzyme B expression increased in trained NK cells (**Figure 1D**). Direct agonistic activation of PKCθ and mobilization of Ca^2+^ by PMA and ionomycin in the preactivated NK cells showed enhanced production of IFN-γ and TNF-α, and cell proliferation, as indicated by Ki67 (**Figure 1D**). These results suggested that trained immunity can be induced by direct activation of intracellular signaling pathways without the involvement of cell surface receptors.

In preactivated NKT cells, similarly to the trained NK cells, IFN-γ production increased in response to cytokine restimulation on day 7 (**Supplementary Figure S1A**); in addition, basal granzyme B levels and PMA plus ionomycin-induced IFN-γ, TNF-α and Ki67 were also significantly enhanced in preactivated NKT cells on day 7 (**Supplementary Figure S1B**), thus indicating a common feature shared by NK and NKT cells in cytokine-induced immune memory.

### CD57- and NKG2A-defined NK subsets show phenotypes of trained immunity

**Figure 2:**
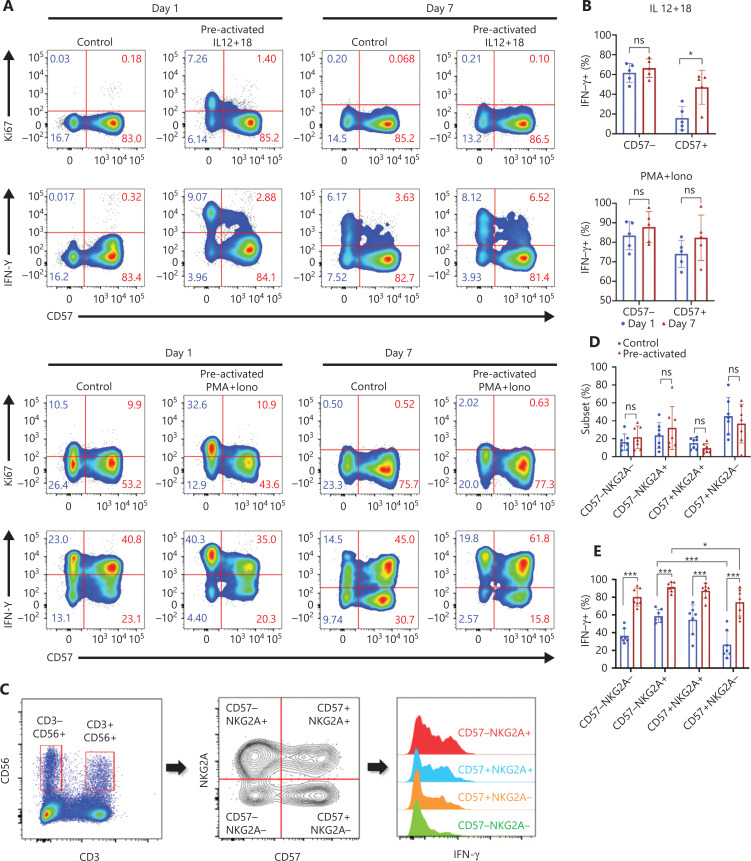
CD57- and NKG2A-defined NK subsets show phenotypes of trained immunity. (A) Upper panel: representative flow cytometry plots of the percentages of Ki67^+^ or IFN-γ^+^ cell populations in NK cells stimulated with IL-12/15/18 for 16 h on day 0 and restimulated with IL-12/18 on day 7; lower panel: PMA+ionomycin restimulated cells on day 7 to obtain positive controls. Data are representative of 3 independent experiments. (B) Upper panel: summary bar graph showing the percentage of IFN-γ^+^ cells in the CD57^−^ and CD57^+^ NK subsets in the upper panel of (A); Lower panel: summary bar graph showing the percentage of IFN-γ^+^ cells in the CD57^−^ and CD57^+^ NK subsets in the lower panel of (A). (C) Flow cytometry plot of the gating strategy for CD3^−^CD56^+^ NK cell subsets. NK cells were stained with NKG2A, CD57, and IFN-γ, and grouped into 4 subsets according to NKG2A and CD57 for IFN-γ expression analysis. (D, E) Summary data showing the percentage of each of the 4 NK cell subsets (D) and the percentage of IFN-γ^+^ NK cells (E) in the control and preactivated groups on day 7 of *in vitro* culture. For all experiments, *n* = 5–7, *P < 0.05, **P < 0.01, and ***P < 0.001 (error bars, mean ± SEM). Data are representative of at least 3 independent experiments.

To study the cellular origin of cytokine-induced trained immunity, we first grouped NK cells according to the presence or absence of the developmental marker CD57 and compared effector features between subsets. On day 1 (1 day after preactivation by IL-12/15/18), more than half the CD57− NK cells showed active proliferation and increased IFN-γ production (**Figure 2A**), whereas the controls had no CD57^−^ cells with effector features. These results suggested rapid activation of CD57^−^ NK cells after IL-12/15/18 preactivation. CD57^+^ NK cells produced much less IFN-γ than CD57^−^ cells on day 1, but IFN-γ production by the CD57^+^ subset was significantly enhanced on day 7 (**Figure 2B**, upper panel), thereby indicating the development of NK cells from CD57^−^ to CD57^+^ during cytokine induction of trained immunity. However, CD57 alone was insufficient to define the precursors of NK cells with trained immunity, because approximately half the CD57^−^ cells did not respond to restimulation. We then included another developmental marker, NKG2A, in the system. NKG2A^−^ and NKG2A^+^ NK cells showed similar levels of effector features after preactivation on day 1 (**Supplementary Figure S2**). When both CD57 and NKG2A are used, CD57^−^NKG2A^+^ NK cells can be considered the “youngest” NK cells among the 4 subsets^11^. We found no significant differences between the control and preactivated groups in terms of secondary responses for each subset on day 7 (**Figure 2C and 2D**). The IFN-γ production ability of NK cells was clearly enhanced by IL-12/15/18 preactivation (**Figure 2C and 2E**); however, this ability was not confined to any of the 4 subsets. The traditional developmental markers for peripheral NK cells, CD57 and NKG2A, were unable to identify the precursors of NK cells with trained immunity, although CD57 appeared to be a better marker than NKG2A.

### scRNA-seq analysis of cytokine-induced trained immunity in NK cells

**Figure 3:**
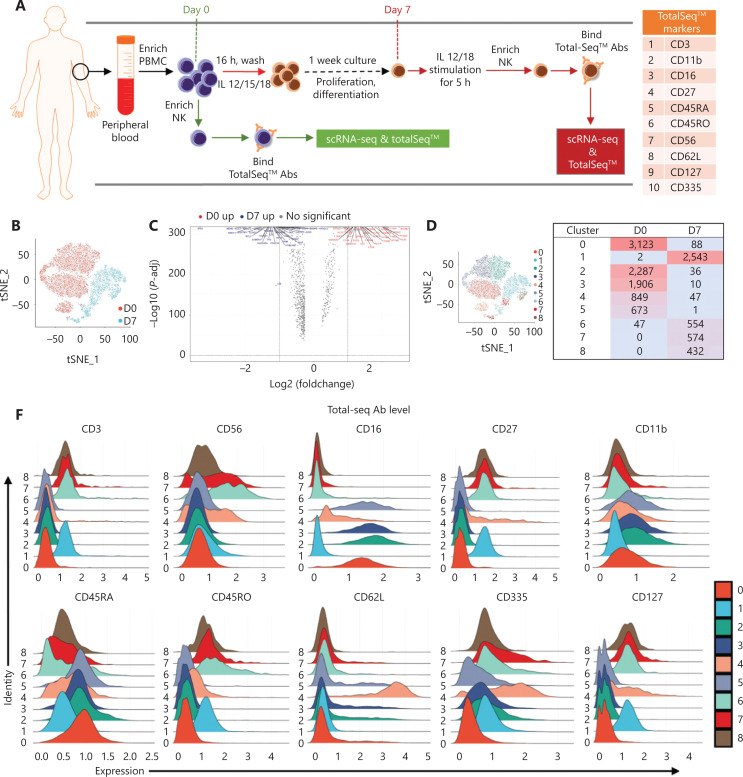

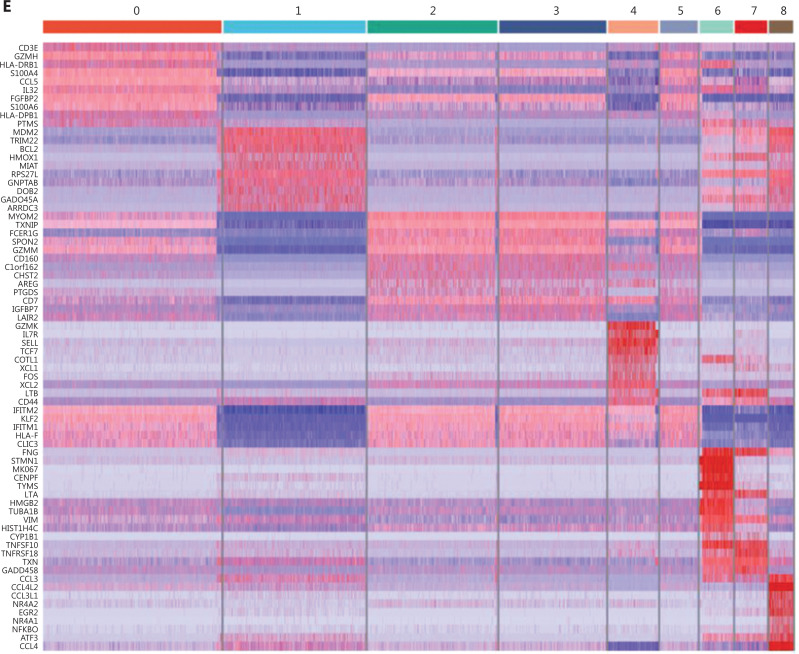
scRNA-seq analysis reveals shifted NK heterogeneity along with the formation of cytokine-induced trained immunity. (A) Scheme of the overall design of the scRNA-seq experiment. PBMCs were isolated from fresh peripheral blood. One-quarter of the PBMCs were used to enrich day 0 NK cells, and the remaining PBMCs were preactivated with IL-15/12/18 for 16 h, then washed and cultured in medium containing a low dose of IL-15 for 1 week. The cells were restimulated with IL-12/18 for 5 h on day 7 and then enriched in day 7 NK cells. The enriched day 0 or day 7 NK cells were labeled with 10 TotalSeq™ antibodies, sequenced and analyzed. The table on the right lists the 10 TotalSeq™ antibodies examined: CD3, CD16, CD56, and CD336 for T and NK lineage definition; CD11b and CD27 for NK cell differentiation; and CD45RA, CD45RO, CD27, and CD62L for T cell memory. (B) *t*-SNE projection of a total of 14,765 NK cells, showing the distribution of day 0 (D0) and day 7 (D7) NK cells. Red indicates day 0, and blue indicates day 7. (C) Volcano plot of differentially expressed genes in NK cells on day 0 and day 7. All genes passing the *P*-value and fold difference thresholds are labeled with colored gene names. (D) 9 main NK clusters were numbered and are displayed with a *t*-SNE plot (left), and details on the composition and cell number for these 9 clusters are shown in the table (right). Day 0 clusters were #0, #2, #3, #4, and #5. Day 7 clusters were #1, #6, #7, and #8. (E) The top 10 upregulated differentially expressed genes (ranked by log fold change) of each cluster were plotted with a heatmap. (F) Overlaid histogram showing the expression profile of the cell surface antigens labeled by 10 TotalSeq™ antibodies in the 9 defined NK cell clusters. CD16, CD11b, and CD45RA were higher in all day 0 clusters; CD3 and CD45RO were higher in all day 7 clusters; CD56 and CD335 were higher in cluster 4# of day 0 and clusters #6 and #7 of day 7; CD27 and CD127 were higher in all day 7 clusters and in cluster #4 of day 0; and CD62 L was very high in cluster #4 of day 0 NK cells.

We then used scRNA-seq and TotalSeq™ oligo-conjugated antibodies to define the precursors of trained NK cells. Human NK cells were collected on day 0 and day 7 post *in vitro* preactivation, incubated with TotalSeq™ antibodies, and then subjected to scRNA-seq with the 10x Genomics platform (**Figure 3A**). NK cells from 3 healthy donors (**Supplementary Table S1**) showed similar levels of cytokine-induced trained immunity, and all contained CD57^+^NKG2C^+^ HCMV-induced memory NK cells (**Supplementary Figure S3A**). We first combined a total of 10,420 day 0 cells and 4,345 day 7 cells for analysis (**Supplementary Figure S3B**). The initial clustering resulted in 11 distinct clusters. Raw clusters #5, #7, #10, and #11 expressed markers for lineages other than NK cells (**Supplementary Figure S3C**). Therefore, we excluded these 4 clusters from further analysis to focus on NK lineage cells.

**Figure 4:**
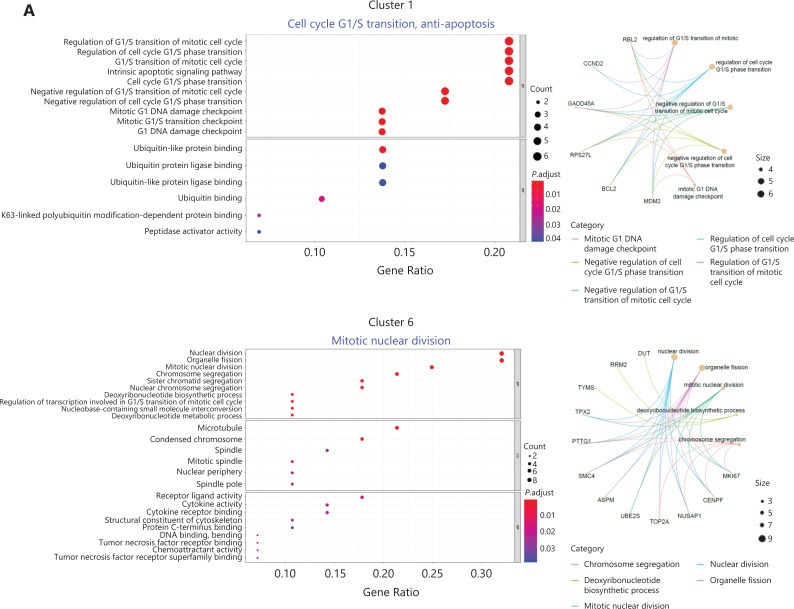

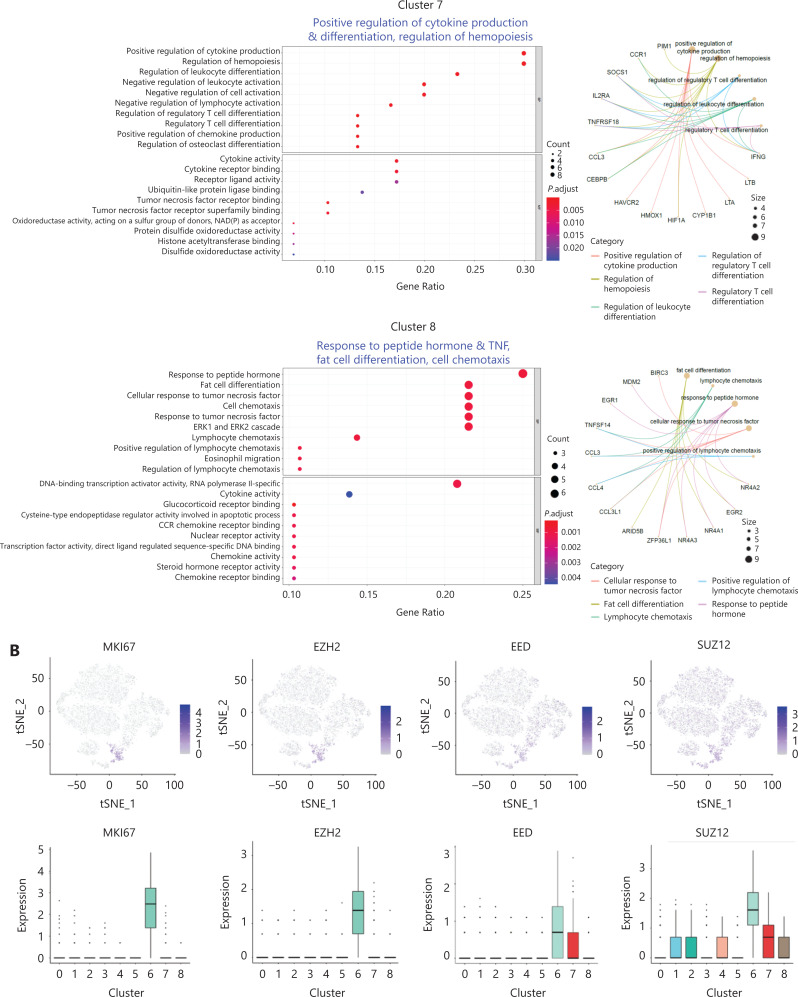

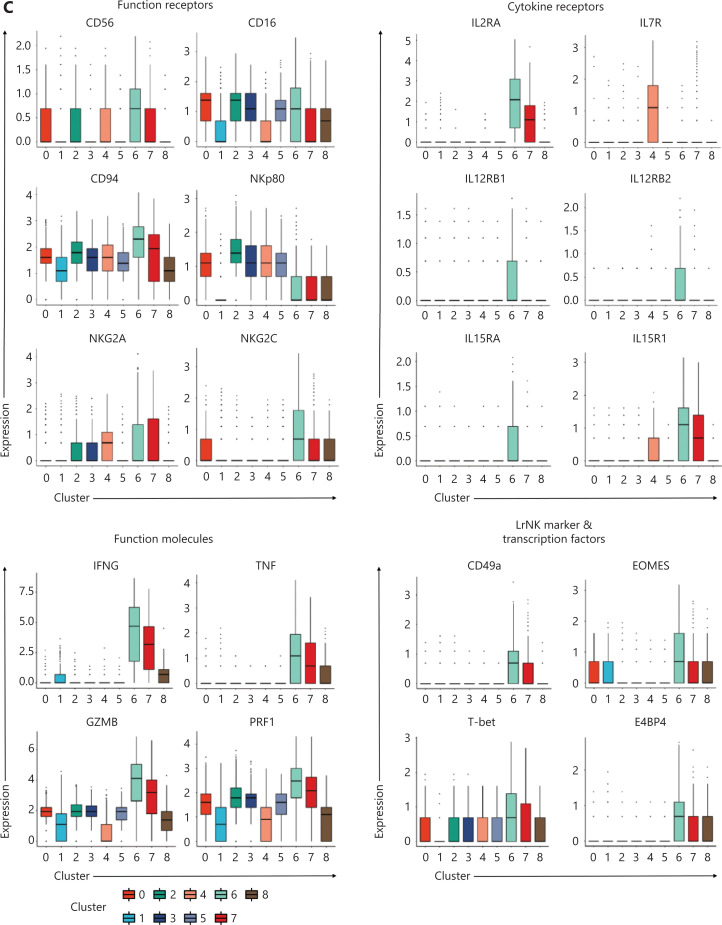
Identification of memory-related clusters in IL-12/15/18-preactivated NK cells. (A) GOEA plots showing active pathways and gene networks enriched in 4 NK clusters from day 7 NK cells. (B) *t*-SNE and box plots demonstrating the expression profiles of MKI67, EZH2, EED, and SUZ12 in all 9 NK clusters. (C) Box plots showing the expression of classified functional genes in 9 NK clusters.

In the remaining clusters, little overlap was observed between day 0 and day 7 NK cells (**Figure 3B**). A volcano plot showed significant gene expression changes between NK cells on day 0 and day 7 (**Figure 3C**), and *IFNG* was the most highly elevated gene in day 7 NK cells (**Figure 3C**), results consistent with the IL-12/15/18-induced trained immunity phenotype. The day 0 and day 7 cells were combined and grouped into 9 clusters that were visualized with a *t-*SNE plot (**Figure 3D**). The top 10 differentially expressed genes in each of the 9 clusters showed distinct features and functions of the NK cell cluster that they represented (**Figure 3E**). Among the 9 clusters, 5 were mainly from day 0 NK cells: cluster #0 expressed *CD3E*,* GZMH*, and *HLA-DRB1* (**Figure 3E**), which showed positive regulation of lymphocyte activation (**Supplementary Figure S3D**); both clusters #2 and #3 expressed *MYOM2*,* FCER1G*, and *SPON2* (**Figure 3E**), which showed positive regulation of the immune effector process (**Supplementary Figure S3D**); cluster #4 expressed *IL7R*,* SELL*, and *TCF7* (**Figure 3E**), which showed features of T cell memory (**Supplementary Figure S3D**); and cluster #5 expressed *IFITM2*,* GZMM*, and *FGFBP2* (**Figure 3E**), which showed a response to the type I interferon signaling pathway (**Supplementary Figure S3D**). According to their gene expression profiles, cluster #0 was identified as adaptive NK cells; cluster #4 was identified as CD56^bright^ NK cells; and clusters #2, #3, and #5 were identified as mature NK cell subsets. However, the 4 major clusters from day 7 NK cells exhibited characteristics that correlated with cytokine-induced trained immunity: cluster #1 expressed *MDM2*,* BCL2*, and *GADD45A* (**Figure 3E**), thus indicating that the cells were arrested in cell cycle progression (**Figure 4A**); cluster #6 expressed *IFNG*,* MKI67*, and *CENPF* (**Figure 3E**), thus indicating that this cluster was in active mitotic division (**Figure 4A**); cluster 7# expressed *IFNG*,* LTB*, and *LTA* (**Figure 3E**), thus indicating that the cluster was involved in hematopoiesis and positive regulation of cytokine production; and cluster #8 expressed *CCL3*,* CCL4*,* CCL3L1*, and *CCL4L2* (**Figure 3E**), thus indicating that the cluster responded to cell chemotaxis (**Figure 4A**). The relationship between day 7 NK cell clusters and cytokine induction of trained immunity is discussed in detail in the following sections.

The expression levels of TotalSeq™ antibody-labeled proteins on the 9 NK clusters displayed different profiles between day 0 and day 7 NK cells (**Figure 3F**). According to the protein expression of these 10 surface markers, some clusters showed featured expression patterns: cluster #4 of day 0 was CD56^hi^CD16^low^ CD27^+^CD11b^+^CD45RA^+^ CD45RO^dim^CD62L^hi^CD335^+^CD127^+^, thus indicating properties resembling those of central memory T cells. In addition, all NK subsets from day 7 were CD3^dim^CD16^−^CD27^+^CD11b^−^CD45RA^dim^CD45RO^hi^CD127^+^CD335^+^, thus showing memory and immature features, with clusters #6 and #7 having higher expression of CD56 (**Figure 3F**). However, we did not observe perfect positive correlations between the protein levels of the 10 markers and their mRNA expression levels (**Supplementary Figure S3E**). The discrepancy between the mRNA levels from scRNA-seq and levels of surface proteins from TotalSeq™ should be cause for concern among the scientific community in the interpretation of complex genomic data.

### Identification of NK subsets that give rise to cytokine-trained immunity

Transcriptome analysis showed that both clusters #6 and #7 of day 7 NK cells highly expressed *IFNG*, but only cluster #6 was *MKI67*-positive and actively proliferating (**Figure 4A**). Cell cycle-concentrated GOEA of cluster #6 further revealed an active mitotic cell cycle state, with* EZH2* at the center of the gene network (**Supplementary Figure S4A**). In addition to *EZH2*, *EED* and *SUZ12*, which encode components of the core PRC2 complex, along with *MKI67*, were highly expressed in cluster #6, and *MKI67* and *EZH2* were expressed only in cluster #6 (**Figure 4B**). In addition, DNA methyltransferase 1 (*DNMT1*), another important epigenetic regulator expressed exclusively in cluster #6, exhibited the same pattern as *MKI67* and *EZH2* (**Supplementary Figure S4B**). This finding is notable because *DNMT1* is required to maintain a progenitor state epigenetically^12^. The expression patterns of these genes indicated that cluster #6 had a distinct epigenetic program that might favor its unique function of initiating trained immunity, because epigenetic rewiring is essential for generating trained immunity.

Next, we examined the expression of a series of functional genes in all 9 NK clusters and revealed the uniqueness of cluster #6. For NK receptors, cluster #6 was high in *CD56*,* CD94*, and* NKG2C*, and low in* KLRF1* (NKp80) (**Figure 4C**, upper left panel). For cytokine receptors, cluster #6 was the only group of cells that expressed *IL2RA*,* IL12RB1*,* IL12RB2*,* IL15RA*, and *IL18R1* together (**Figure 4C**, upper right panel). The receptors encoded by these genes enable NK cells to respond to the cytokines IL-2, IL-12, IL-15 and IL-18, all which are essential for the development and function of NK cells and for the induction of trained immunity of NK cells. Cluster #6 expressed the highest levels of NK effector genes, including *IFNG*,* TNF*,* GZMB*, and *PRF1*, followed by cluster #7 (**Figure 4C**, lower left panel). Suppressor of Cytokine Signaling 1 (*SOCS1*) was also highly expressed in clusters 6# and 7#, thereby indicating that negative feedback regulation for cytokine signal transduction had been evoked. In addition, *CD2*, encoding a cell adhesion molecule that functions as a costimulatory receptor in HCMV-induced memory NK cells^13^, was expressed at the highest level in cluster #6 (**Supplementary Figure S4B**). Hapten-induced memory NK cells in a mouse model have been found to be of liver origin^14^; therefore, we examined the expression of markers for liver-resident NK cells in cluster #6. *ITGA1* (CD49a), *EOMES*, and *TBX21* (T-bet), which are markers for liver-resident NK cells^15,16^, were all expressed in cluster #6. The transcription factor gene *E4BP4*, which is important for NK cell development^17^, was also highly expressed in cluster #6 (**Figure 4C**, lower right panel), thus indicating the possibility of a liver origin of cytokine-trained NK cells in humans. This unique combination of gene expression indicated that cluster #6 might contain the cells that give rise to trained immunity in NK cells.

### Developmental pathway of cytokine-trained NK cells

**Figure 5:**
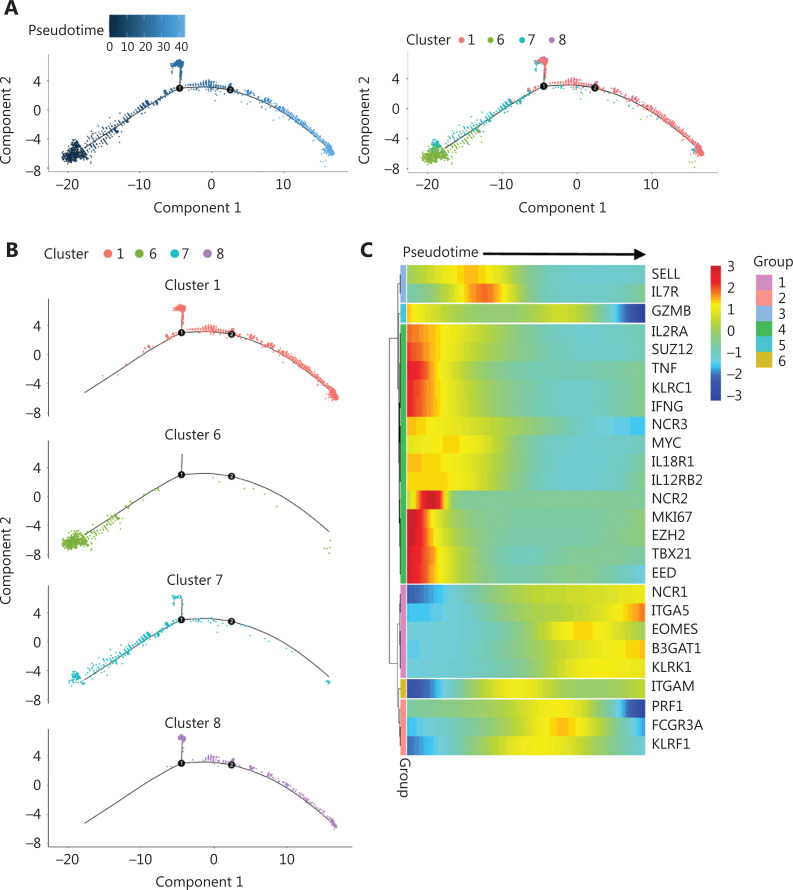
Trajectory analysis reveals the developmental pathway of cytokine-trained NK cells. (A) Analysis of day 7 NK cells with Monocle2 pseudotime trajectory analysis to simulate the ontology (left) and positions of cells in all 4 clusters (right). (B) Individual clusters of day 7 NK cells are demonstrated in the trajectory. (C) Heatmap of selected gene expression profiles along the pseudotime trajectory. Group 1 genes, including *B3GAT1*,* ITGA5* (*CD49e*), and* KLRK1* (*NKG2D*), were more highly expressed at the late developmental stage. Group 2 genes, including *PRF1*,* FCGR3A* (*CD16*), and* KLRF1* (*NKp80*), were more highly expressed at the late-middle stage. Group 3 genes, including *SELL* (*CD62 L*) and* IL7R*, were more highly expressed at the early-middle stage of development. Group 4 genes were expressed at an early stage of the developmental pathway, which was cluster #6. The group 5 gene *GZMB* showed higher expression at the initial and late developmental stages, and the group 6 gene *ITGAM* (*CD11b*) showed higher expression at the middle and late stages of development.

Because of its features, we reasoned that cluster #6 might contain the precursor of cytokine-trained NK cells. A pseudotime trajectory analysis of day 7 NK cells showed a relative linear developmental progression with only 2 small branches. Cells in cluster #6 were located exclusively at the “starting” end of the progression trajectory (**Figure 5A**). Clusters #7, #8, and #1 were positioned at later developmental stages than cluster #6, according to pseudotime trajectory analysis (**Figure 5B**).

To further investigate the developmental pathway of cytokine-trained NK cells, we analyzed the expression profiles of genes along the pseudotime trajectory. The genes were classified into 6 groups according to their timing of expression (**Figure 5C**). The heatmap clearly showed that group 4 genes, including* MKI67*,* IFNG*,* TBX21* (*T-bet*),* TNF*,* KLRC1* (*NKG2A*),* IL2RA*, *EZH2*, *EED*, and *SUZ12*, were concentrated at the very beginning of the developmental pathway, which was the position of cluster #6 on the pseudotime trajectory. The results revealed that this gene expression profile may be used as a marker of the “precursor” state for cytokine-induced memory-like NK cells.

### Intrinsic proliferation ability of NK cells correlates with EZH2 expression

Our scRNA-seq results identified EZH2 as a distinctive epigenetic marker of precursors of cytokine-induced memory-like NK cells. Our previous research has indicated that EZH2 is an important regulator of NK lineage commitment and function, and its expression correlates with cell proliferation^10^. Because epigenetic rewiring is essential for trained immunity^7,18^, we studied whether EZH2 might be involved in cytokine induction of trained immunity in NK cells.

**Figure 6:**
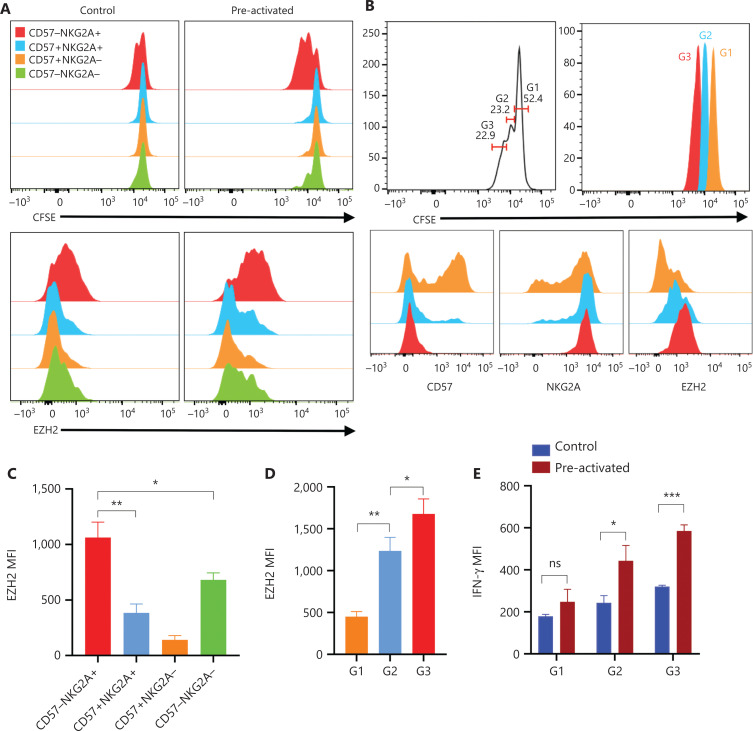
Intrinsic proliferation ability of NK cells correlated with EZH2 expression. (A) PBMCs were labeled with CFSE and cultured *in vitro* under control or preactivation conditions. The upper panel shows overlaid histograms of one representative experiment of the CFSE dilution profile in all 4 NK cell subsets on day 3. The lower panel shows representative overlaid histograms of the expression profile of EZH2 in the same cells as in the upper panel. (B) Upper panel: representative histogram of 3 generations of CFSE-labeled NK cells in PBMCs cultured *in vitro* on day 5 post preactivation with IL-12/18; lower panel: representative overlaid histogram of the expression of CD57, NKG2A, and EZH2 in the first 3 generations of NK cells. (C, D) Summary bar graph showing the expression levels of EZH2 in all 4 indicated NK cell subsets (C) and in the first 3 generations of NK cells (D) on day 3 and day 5 after preactivation with IL-12/18. (E) CFSE-labeled PBMCs were cultured *in vitro* under control or preactivation conditions, restimulated with IL-12/18 on day 5, and assayed for cell division and IFN-γ production. The MFI of IFN-γ in each generation of NK cells from the control and preactivation groups is shown as a summary bar graph. For all experiments, *n* = 5, *P < 0.05, **P < 0.01, and ***P < 0.001 (error bars, mean ± SEM). Data are representative of at least 3 independent experiments.

The scRNA-seq results indicated that the “precursors” of trained NK cells have enhanced effector functions, are sensitive to cytokine stimulation, and possess active proliferation ability. Therefore, we traced the cell divisions of NK cells after cytokine induction. On day 3, the CD57^−^NKG2A^+^ NK subset was the fastest proliferating population (**Figure 6A**, upper panel, right). These results indicated that this subset has a higher intrinsic proliferation ability than the other subsets, because in the control condition of low-dose IL-15, the CD57^−^NKG2A^+^ NK subset also replicated slightly more rapidly than all other subsets (**Figure 6A**, upper panel, left). Intracellular staining of the EZH2 protein revealed that its expression was highest in the CD57^−^NKG2A^+^ NK subset (**Figure 6A**, lower panel, left). After IL-12/15/18 stimulation, the expression of EZH2 was further increased in the CD57^−^NKG2A^+^ NK subset (**Figure 6A**, lower panel, right **and Figure 6C**), an effect correlating with the strongest proliferation ability of this subset.

To further investigate the correlation between cell proliferation and EZH2 expression, we compared the expression of CD57, NKG2A, and EZH2 in each of the first 3 cell divisions/generations on day 5 after IL-12/15/18 stimulation (**Figure 6B**, upper panel). Indeed, the fastest replicating 3rd generation of NK cells expressed the lowest level of CD57 but the highest levels of NKG2A and EZH2 (**Figure 6B**, lower panel **and Figure 6D**), thus further confirming the correlation between EZH2 and the cell cycle in cytokine-trained NK cells. In addition, the 3rd generation of NK cells showed the strongest IFN-γ expression (**Figure 6E**). In agreement with previous scRNA-seq analyses, these results all suggested a strong correlation between EZH2 expression and cytokine-induced trained immunity of NK cells.

### EZH2 promotes the formation of cytokine-induced trained NK cells

**Figure 7:**
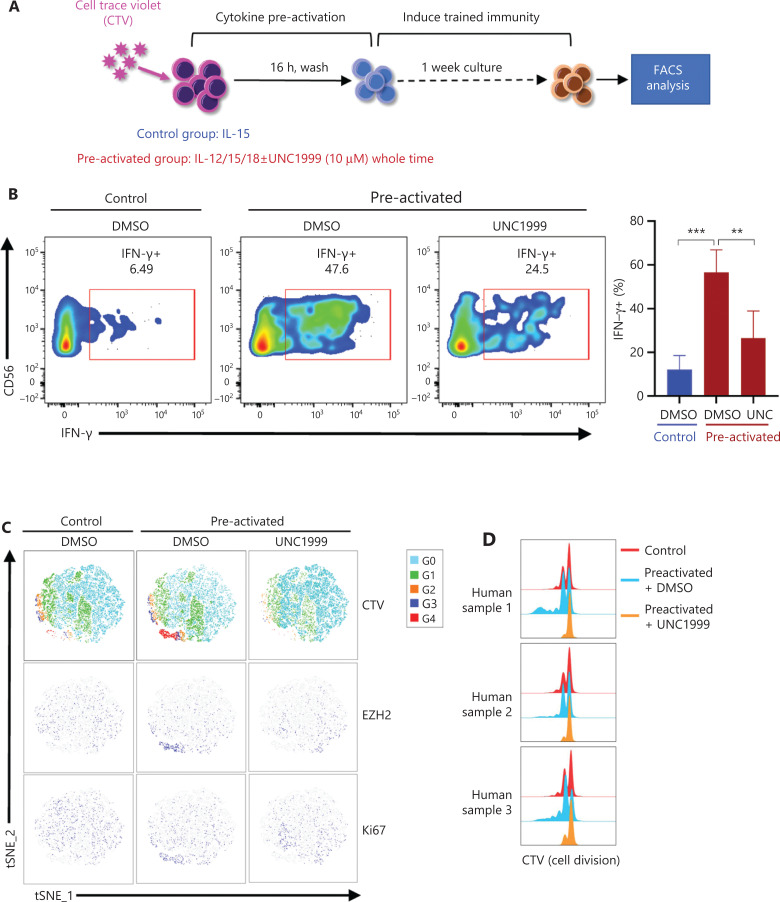
Inhibition of EZH2 represses the formation of cytokine-induced trained NK cells and cell cycle progression. (A) PBMCs were labeled with CTV and cultured *in vitro* under control or preactivation conditions with or without UNC1999 treatment for 1 week, and then the cells were restimulated with IL-12/18. NK cells were analyzed for IFN-γ expression. (B) Representative flow cytometry plot of IFN-γ^+^ NK cells in the control and preactivated groups cultured with or without UNC1999 (left). A summary bar graph shows the percentage of IFN-γ^+^ NK cells in each indicated treatment group (right, *n* = 5). (C) Combined flow cytometry *t*-SNE plot of the CTV dilution and expression of EZH2 and Ki67 in NK cells of each indicated treatment group (*n* = 3). (D) Overlaid histograms show the CTV dye dilution profiles of samples analyzed in the CTV section of (C) (*n* = 3).

The mRNA expression levels of EZH2, EED, and SUZ12 were observed in IFNG^+^MKI67^+^ cluster 6# NK cells (**Figure 4B**), which was set as the developmental origin of cytokine-trained NK cells by pseudotime trajectory analysis (**Figure 5**). EZH2 was expressed in the actively proliferating CD57^−^NKG2A^+^ NK cell subset after IL-12/15/18 preactivation (**Figure 6**). Together, this evidence suggests that EZH2 has a role in the formation of cytokine-trained NK cells, which is associated with cell proliferation. Because of the rarity of EZH2^+^ NK cells in total fresh NK cells, and the limited amount of blood that we were able to obtain from donors, sorting EZH2^+^ NK cells with surrogate surface markers for study was difficult. To demonstrate the necessity of EZH2 during the formation of cytokine-trained NK cells we used UNC1999, a small-molecule inhibitor of EZH2, to treat NK cells during the formation of cytokine-trained NK cells (**Figure 7A**). UNC1999 significantly decreased IFN-γ production, thereby indicating that EZH2 inhibition impaired the formation of cytokine-induced trained immunity of NK cells (**Figure 7B**).

To investigate the relationship between cell cycle progression and EZH2 in the cytokine-induced trained immunity of NK cells, we labeled NK cells with CellTrace Violet (CTV) and then treated cells with UNC1999, IL-12, and IL-18 to inducea trained immunity of NK cells (**Figure 7A**). Representative results showed that the UNC1999-treated preactivated group had no CTV gradient segregation, as compared with the control and untreated preactivated groups, thus indicating that cell cycle progression was blocked (**Figure 7C**). In agreement with previous observations, EZH2 was expressed primarily in the actively proliferating NK cluster, which was the 4th generation, as indicated by the CTV peak (**Figure 7C**). A merged comparison of CTV-labeled cell division peaks clearly showed the differences among the 3 groups: the preactivated group divided more than the other 2 groups, and the UNC1999-treated preactivated group almost stopped dividing (**Figure 7D**). EZH2^+^ NK cells rapidly proliferated during the development of cytokine-trained NK cells.

The EZH2^+^ NK cell subset (cluster #6) was the only NK cell subset expressing the cytokine receptors IL-2R, IL-12R, IL-15R, and IL-18R. IL-12/15/18 stimulation activated EZH2^+^ NK cells, thus leading to their rapid proliferation and subsequent differentiation into cytokine-trained NK cells (**Supplementary Figure S5**). Inhibition of EZH2 methyltransferase activity may block the proliferation of the EZH2^+^ NK cell subset and finally prevent their progeny from differentiating into cytokine-trained NK cells (**Supplementary Figure S5**).

## Discussion

We investigated the mechanisms underlying the formation of cytokine-induced trained immunity of NK cells. We first demonstrated that primary human NK cells preactivated by IL-12/15/18 were able to gain trained immunity with enhanced effector functions, results consistent with the memory-like phenotype of NK cells found by others^6,8^. We then found that traditional developmental markers for NK, CD57, and NKG2A were unable to define the precursors of trained NK cells.

To further study NK cell heterogeneity during the formation of cytokine-induced trained immunity and to trace precursors of trained NK cells, we performed scRNA-seq plus TotalSeq™ on cytokine-trained human NK cells at day 0 and day 7. Unbiased clustering showed that the NK cell transcriptome and clusters changed significantly before and after memory formation. Analysis of 10 TotalSeq™ markers showed that all day 7 NK cells were CD3dimCD16^−^CD27^+^CD11b^−^CD45RA^dim^CD45RO^hi^CD127^+^CD335^+^. For human NK cells, the CD11b^−^CD27^+^ NK subset is from the cord blood and shows the highest ability to secrete cytokines^19^; therefore, this group of NK cells on day 7 had the properties of immature NK cells. CD45RA^dim^CD45RO^+^CD127^+^ is a common feature of memory T cells and consequently may have memory features. Elevated CD335 (NKp46/NCR1) expression indicated enhanced NK cell lytic activity. Therefore, NK cells proliferated and differentiated to a state with features of enhanced effector functions, memory T cells and immature NK cells after preactivation with IL-12/15/18.

Transcriptomic analysis and GOEA identified a distinct CD57^−^NKG2A^+^EZH2^+^IFNG^+^MKI67^+^IL12R^+^IL15R^+^IL18R^+^ NK cell cluster #6 on day 7, which was in mitosis phase. Further examination of gene expression showed that cluster #6 (1) expressed *EZH2*,* EED*, *SUZ12*, and *DNMT1*; (2) expressed high levels of *CD56*,* CD94*,* NKG2C*, and *CD2* and low levels of *NKp80*; (3) expressed cytokine receptors for IL-2, IL-12, IL-15, and IL-18, and had high levels of the functional molecules *IFNG*,* TNF*,* GZMB* and *PRF1*; and (4) had liver-resident NK cell characteristics, e.g., CD49a^+^ and EOMES^high^. A pseudotime trajectory analysis of all clusters in day 7 NK cells further demonstrated that cluster #6 was the earliest stage of cytokine-trained NK cell formation. These results indicated that cluster #6 had features of precursors of trained NK cells.

According to scRNA-seq analysis, EZH2 is a marker identifying the subset of precursors of cytokine-trained NK cells. EZH2 plays a major role in hematopoiesis by promoting pluripotency maintenance and self-renewal of adult stem cells^20–22^. EZH2 is essential for lymphopoiesis and is strongly expressed in proliferating cells, such as human germinal center B cells, cycling T and B lymphocytes, and plasmablasts^23,24^. Moreover, our previous work has revealed the critical role of EZH2 in NK cell lineage commitment and function^10^. To further investigate the role of EZH2 in the trained immunity of NK cells, we examined the expression and function of EZH2 during cytokine-trained NK cell formation with flow cytometry. The expression of EZH2 was heterogeneous and was highest in the actively proliferating CD57−NKG2A^+^ subset; moreover, the expression of EZH2 correlated with the proliferation of NK cells. In addition, the actively proliferating NK cell population, which was the latest cell division generation, had the most pronounced enhanced memory phenotype. Inhibition of EZH2 enzymatic activity impaired cell proliferation and trained NK cell formation after cytokine induction. Although ectopic overexpression of EZH2 in NK/T-cell lymphoma confers a growth advantage independent of histone methyltransferase activity^25^, during the formation of cytokine-trained NK cells, the enzymatic activity of EZH2 is necessary to maintain cell proliferation. Together, these results indicated a correlation between EZH2 and the cell cycle and cytokine-trained immunity generation in NK cells.

In an MCMV infection-induced murine memory NK cell model, typical expansion and contraction phases of NK cells occurred in response to MCMV infection before the generation of MCMV-specific memory NK cells^26^. Therefore, the actively proliferating CD57^−^NKG2A^+^EZH2^+^ NK cell subset identified herein may be responsible for the “expansion” in cytokine-induced memory-like NK cell formation. According to our results, we propose a model of EZH2^+^ cluster #6 NK cell activation during the formation of cytokine-trained NK cells (**Supplementary Figure S5**).

## Conclusions

Our work defined a rare CD57^−^NKG2A^+^EZH2^+^IFNG^+^ MKI67^+^IL12R^+^IL15R^+^IL18R^+^ human NK cell subset as the main origin of cytokine-induced trained immunity in NK cells and additionally identified the critical role of EZH2 in the formation of trained NK cells. The potential for developing EZH2 as a drug target to manipulate trained NK cells in tumor immunotherapy applications is an important issue that must be further explored.

## Data Availability

The accession number for scRNA-seq and TotalSeq™ data reported herein is GSE158349. For original data and scripts, please contact yinjie2015@tmu.edu.cn.
