# Supplementary material for: EZH2 identifies the precursors of human natural killer cells with trained immunity
Source: Cancer Biol Med. 2021 Sep 24;18(4):1021–39. doi: 10.20892/j.issn.2095-3941.2020.0791 (PMC8610163; doi:10.20892/j.issn.2095-3941.2020.0791)
Supplement: Supplementary file 1 [file cbm-18-1021-s001.pdf]

Supplementary materials

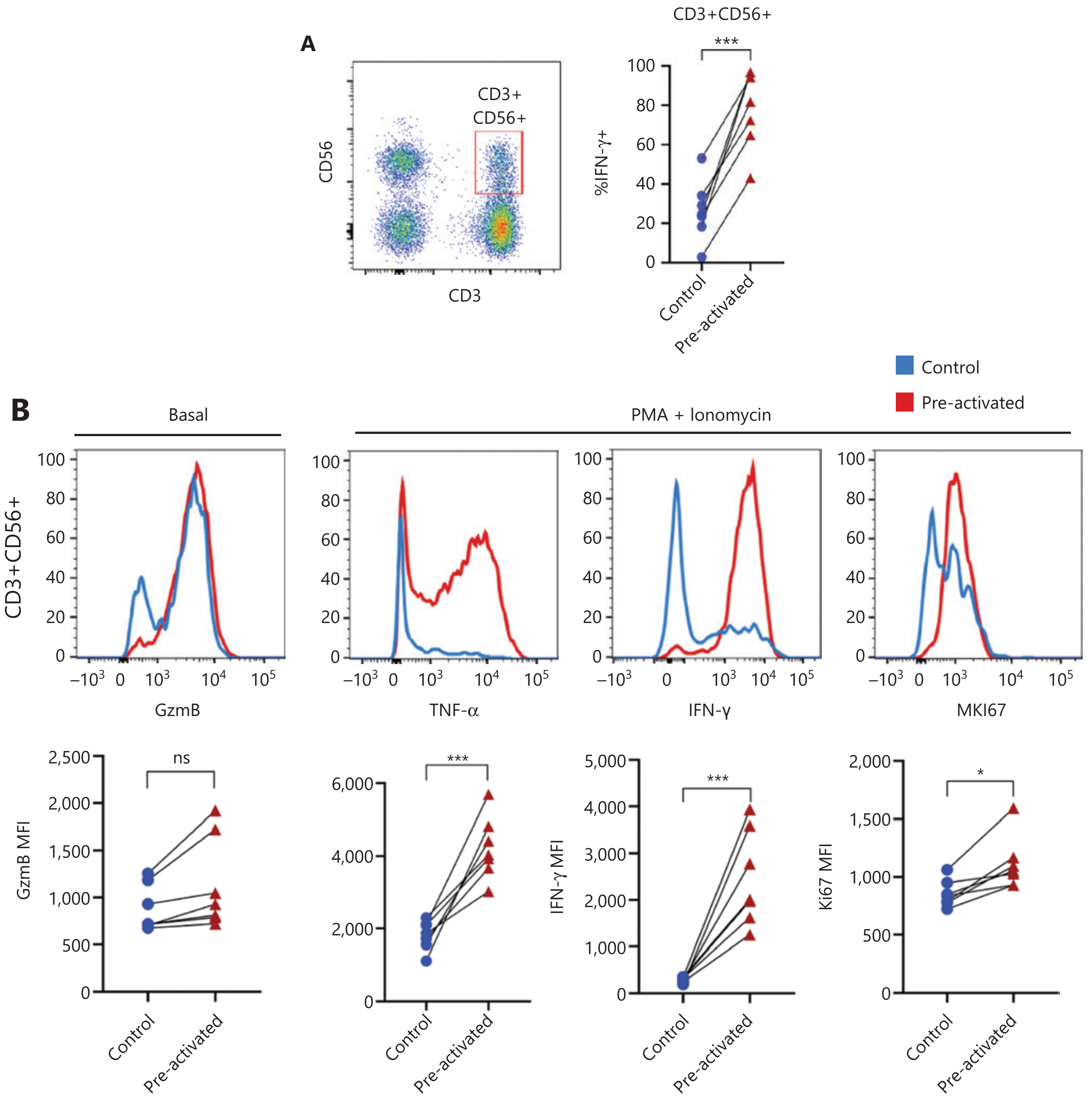

Figure S1 Enhanced effector responses in cytokine trained NKT cells. (A) Left, flow cytometry plot shows the gating strategy for $CD3^+CD56^+$ NKT cells. Right, enhanced IFN-γ production by cytokine trained NKT cells re-stimulated with IL-12/18. (B) Upper panel, representative flow cytometry data showing basal level granzyme B (GzmB), and PMA+Ionomycin stimulated TNF-α, IFN-γ and Ki67 expression in cytokine trained NKT cells compared with control NKT cells. Lower panel is summary data showing the median fluorescence intensity (MFI) of GzmB, TNF-α, IFN-γ and Ki67. For all experiments, n = 7, *P < 0.05, **P < 0.01, and ***P < 0.001 (error bars, mean ± SEM). Data are representative of at least three independent experiments.

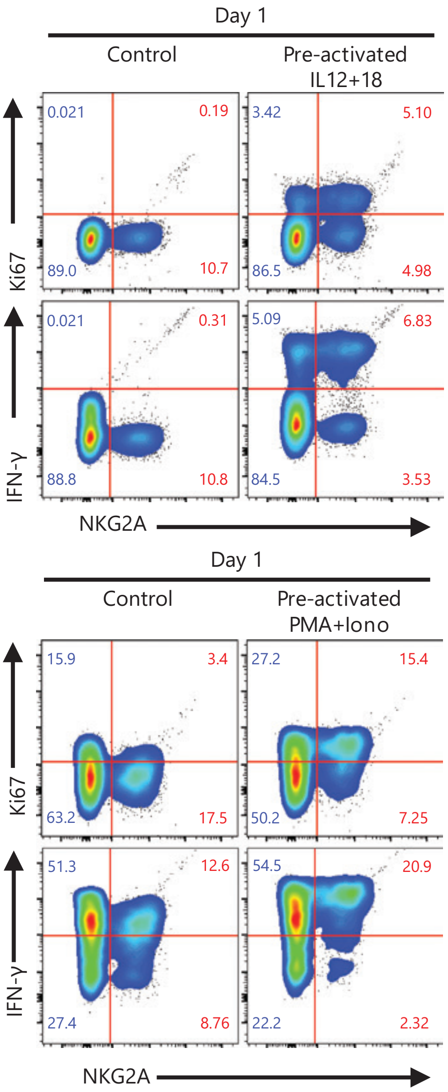

Figure S2 Phenotypes of trained immunity in NKG2A defined NK subsets. Representative flow cytometry plots showing percentage of Ki67^{+} or IFN-γ^{+} cell populations in NKG2A^{-} and NKG2A^{+} NK subsets stimulated with IL-12/18 or PMA+Ionomycin on Day 1. Data are representative of three independent experiments.

A

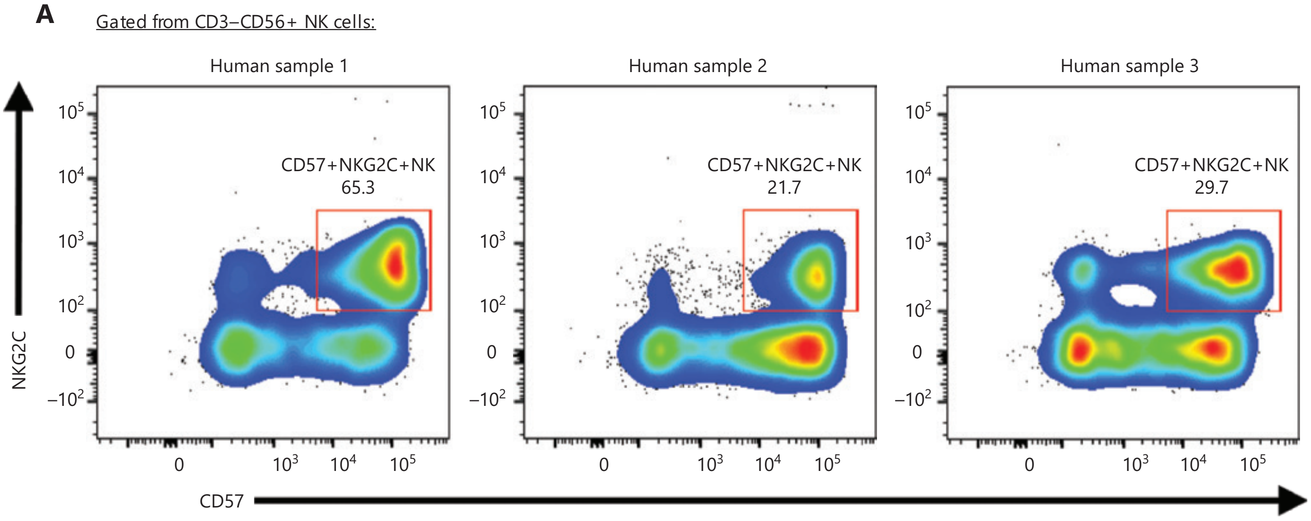

B

	Cell count raw	Cell count filtered	Median UMI count raw	Median UMI count filtered	Median gene count raw	Median gene count filtered	Median percent MT raw	Median percent MT filtered
Human sample 2_day 0	10,833	10,420	3,803	3,833.5	1,524	1,533	6.024465	5.950564
Human sample 2_day 7	4,721	4,345	4,026	4,270	1,738	1,809	2.131638	2.003688

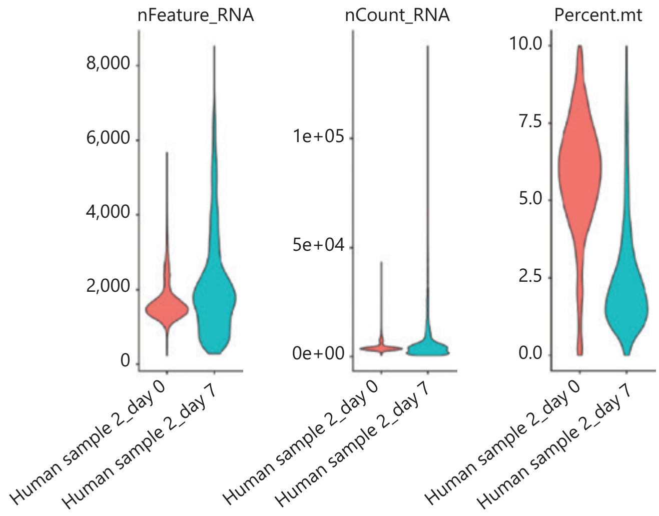

Figure S3 Continued

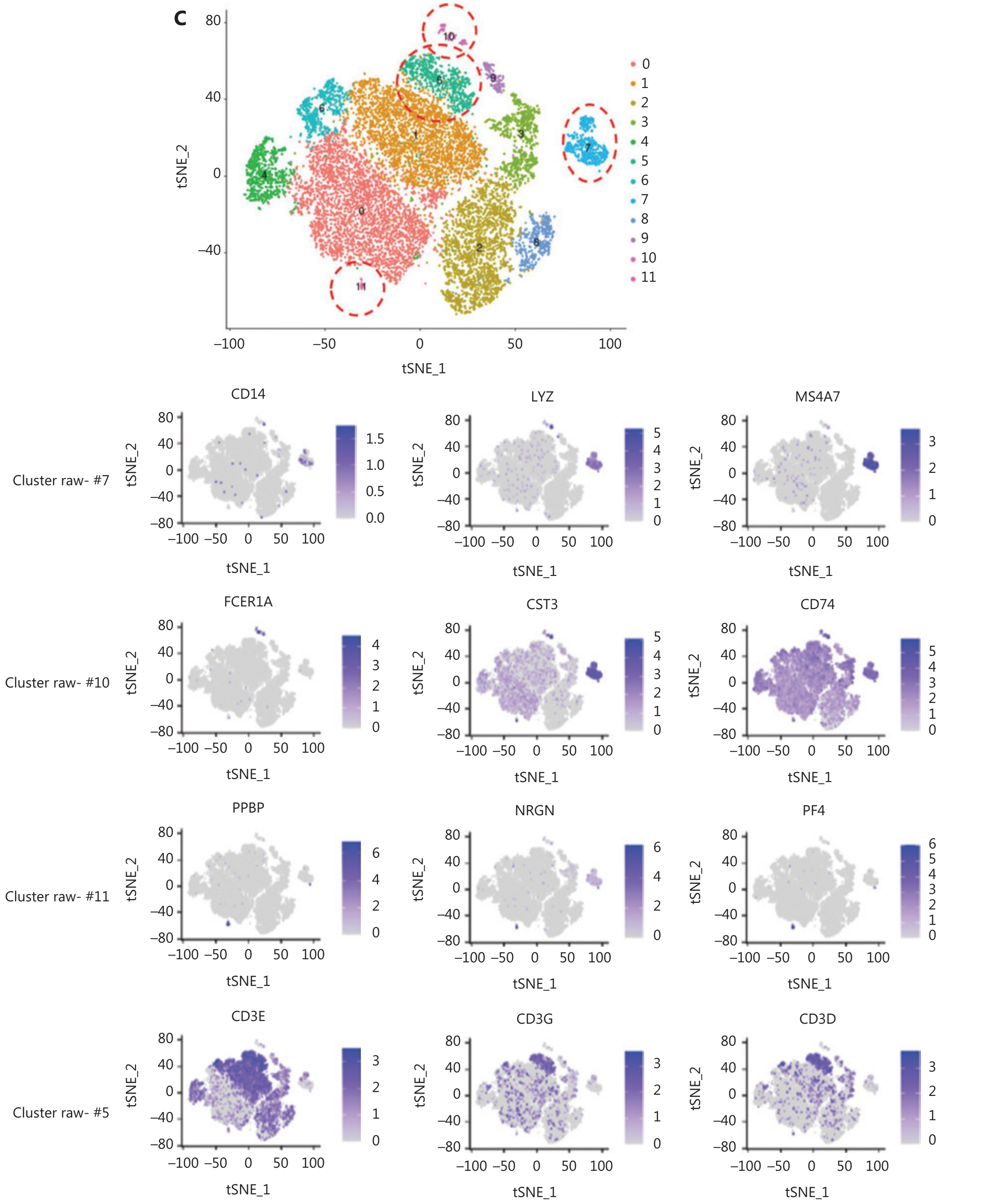

Figure S3 Continued

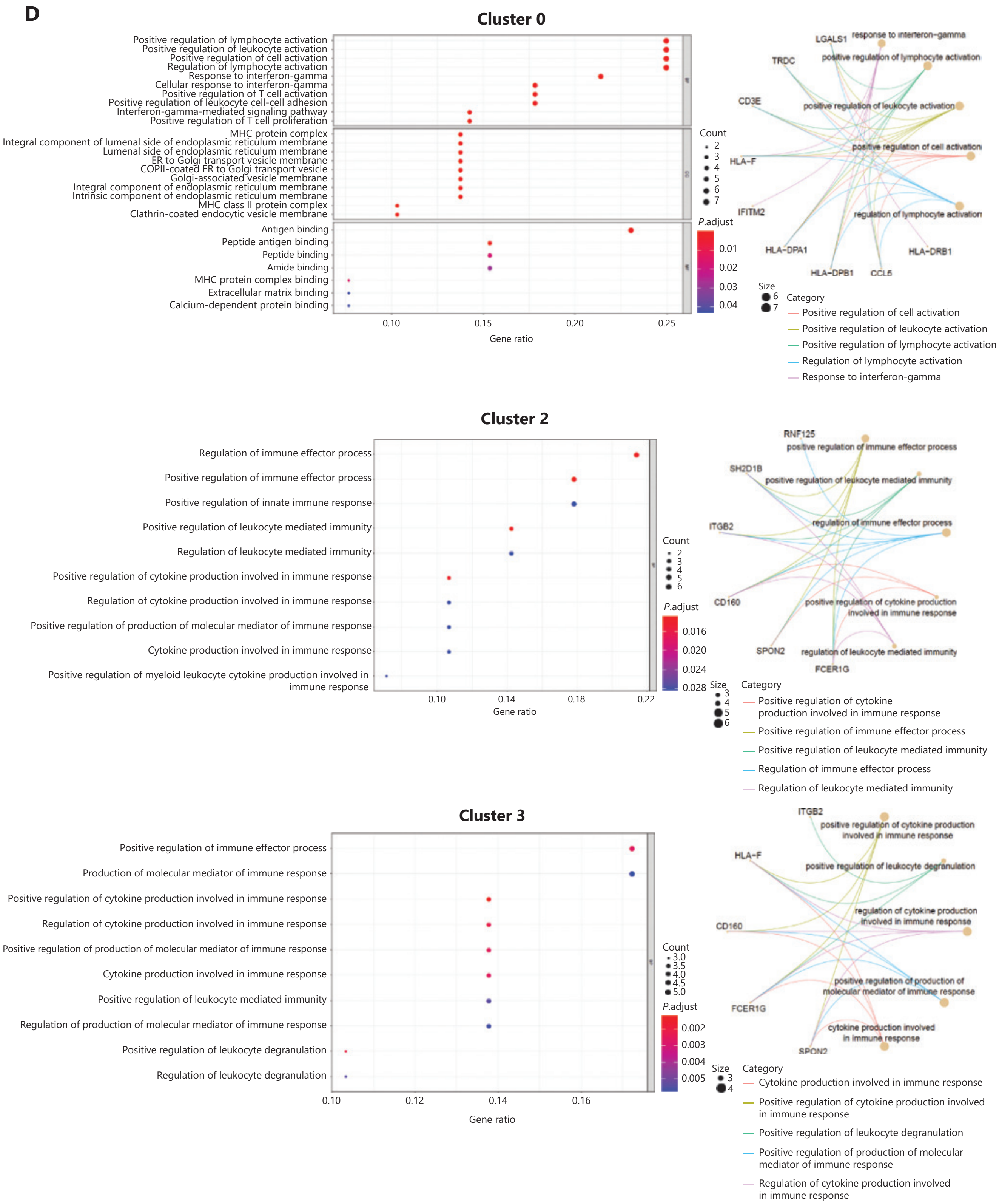

Figure S3 Continued

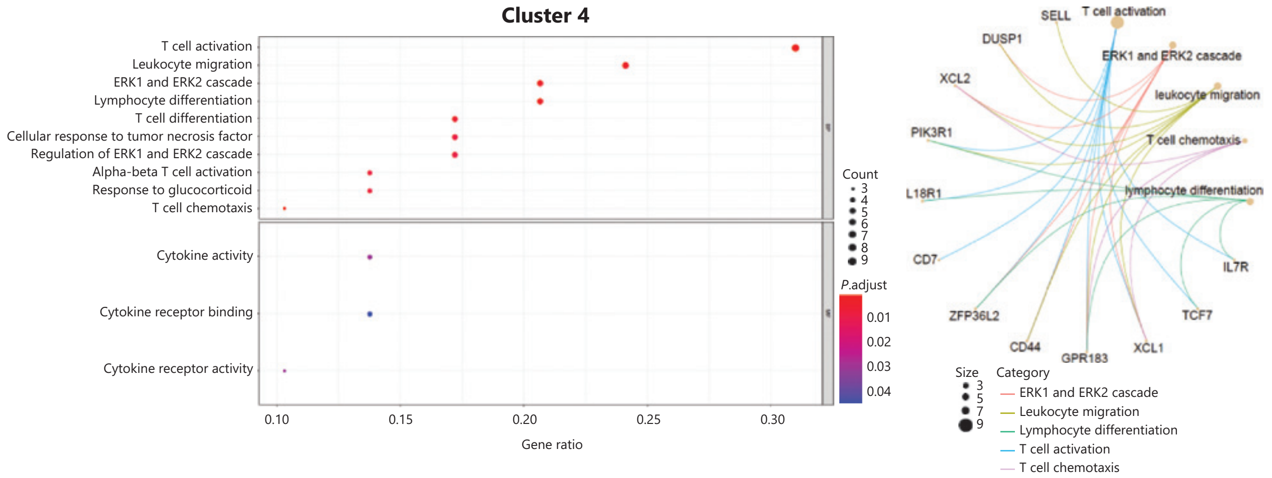

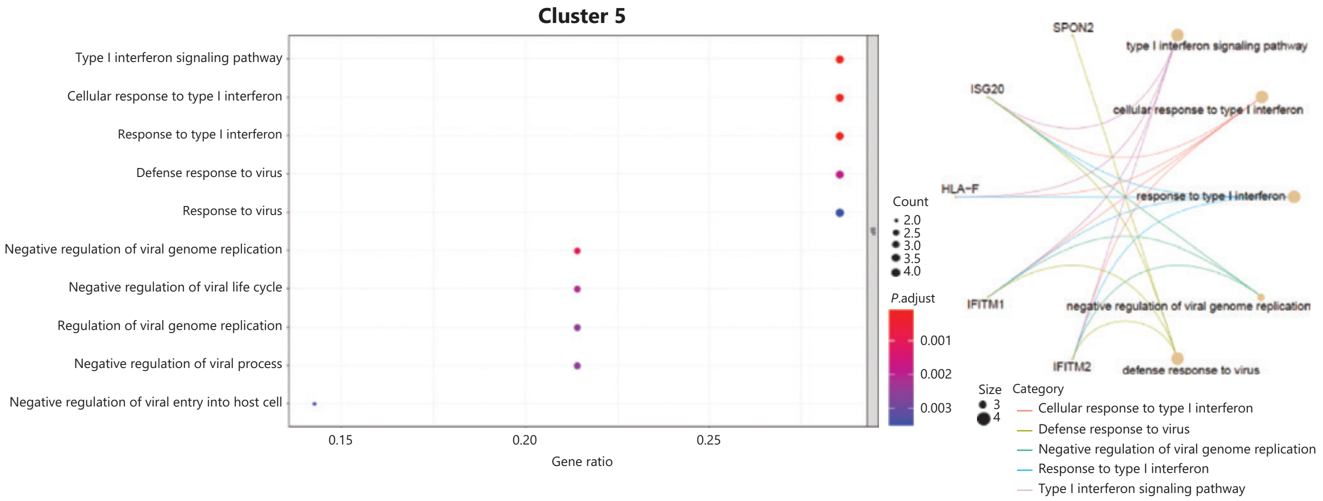

Figure S3 Continued

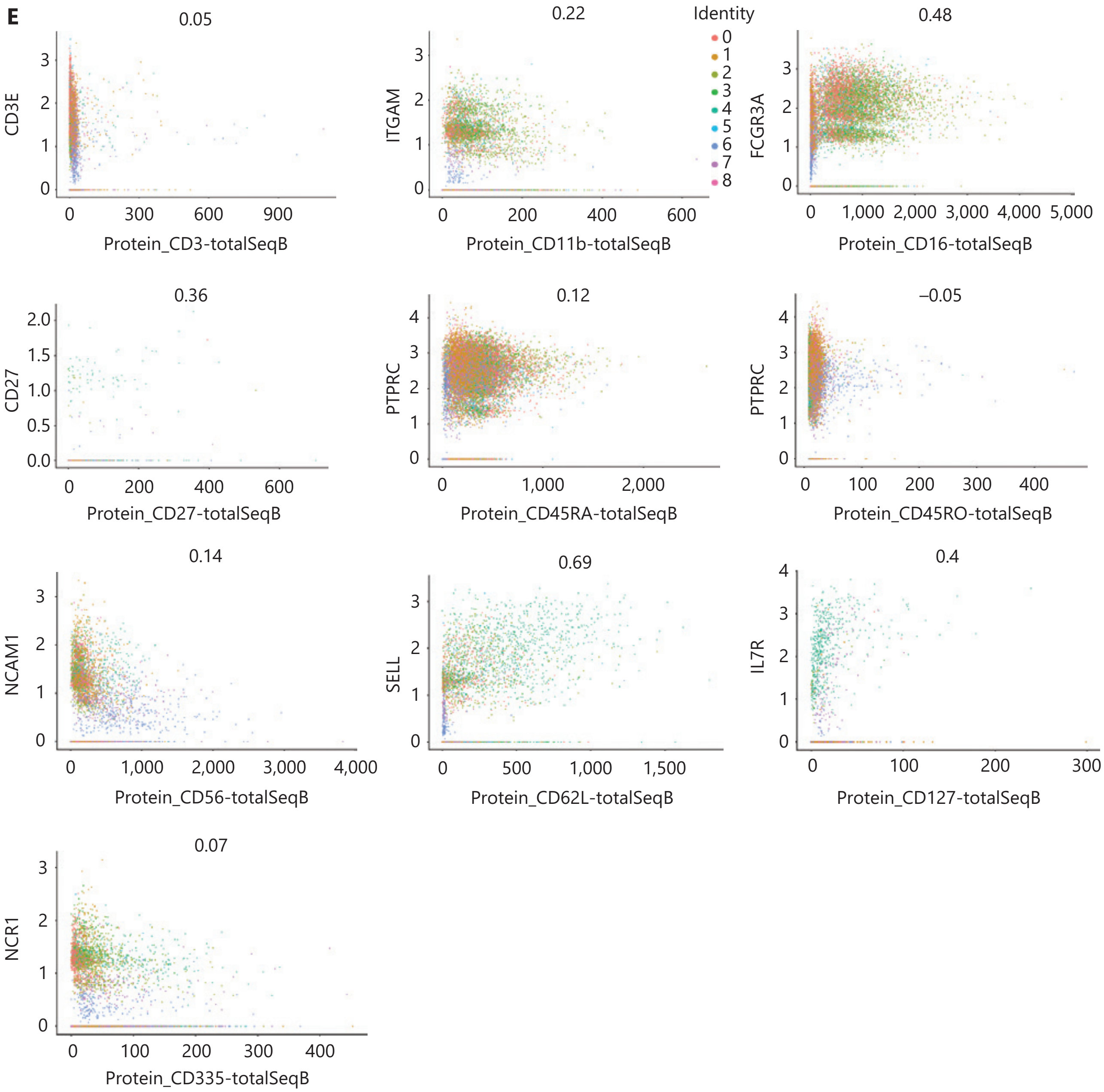

Figure S3 Results associated with NK cell scRNA-seq and TotalSeq™. (A) Flow cytometry plot shows the percentage of $CD57^+NKG2C^+$ HCMV induced memory NK cells in three PBMCs samples selected for scRNA-seq and TotalSeq™. (B) The scRNA-seq and TotalSeq™ data of human sample 2 passed quality control and submitted to analysis. The qualified scRNA-seq samples revealed high cell counts, more than 3,500 median unique molecular identifiers (UMIs) and a minimum of 1,500 genes associated with the cell barcodes. Most of the cells had < 6% of the total gene expression transcribed from mitochondrial genes indicating robust cell viability. (C) After the QC filtering, a total of 10,420 Day 0 NK cells and 4,345 Day 7 NK cells were combined for cluster analysis. The *t*-SNE plot shows 11 raw clusters of all Day 0 and Day 7 NK cells (labeled as raw- #number). There were four clusters highly expressing lineage genes other than NK cells, with cluster raw- #5 expressing T cell-specific markers (*CD3D/E/G*), cluster raw- #7 expressing monocyte-specific markers (*CD14/LYZ/MS4A7*), cluster raw- #10 expressing dendritic cell-specific markers (*FCER1A/CST3*) and cluster raw- #11 expressing platelet markers (*PPBP/NRGN/PF4*). Raw cluster #5, #7, #10 and #11 were excluded for further analysis. (D) GOEA plots show active pathways and gene networks enriched in NK clusters #0, #2, #3, #4 and #5. (E) 2D scatter plots show the interrelationship between a gene's protein and mRNA expression quantified by TotalSeq™TM and scRNA-seq, respectively.

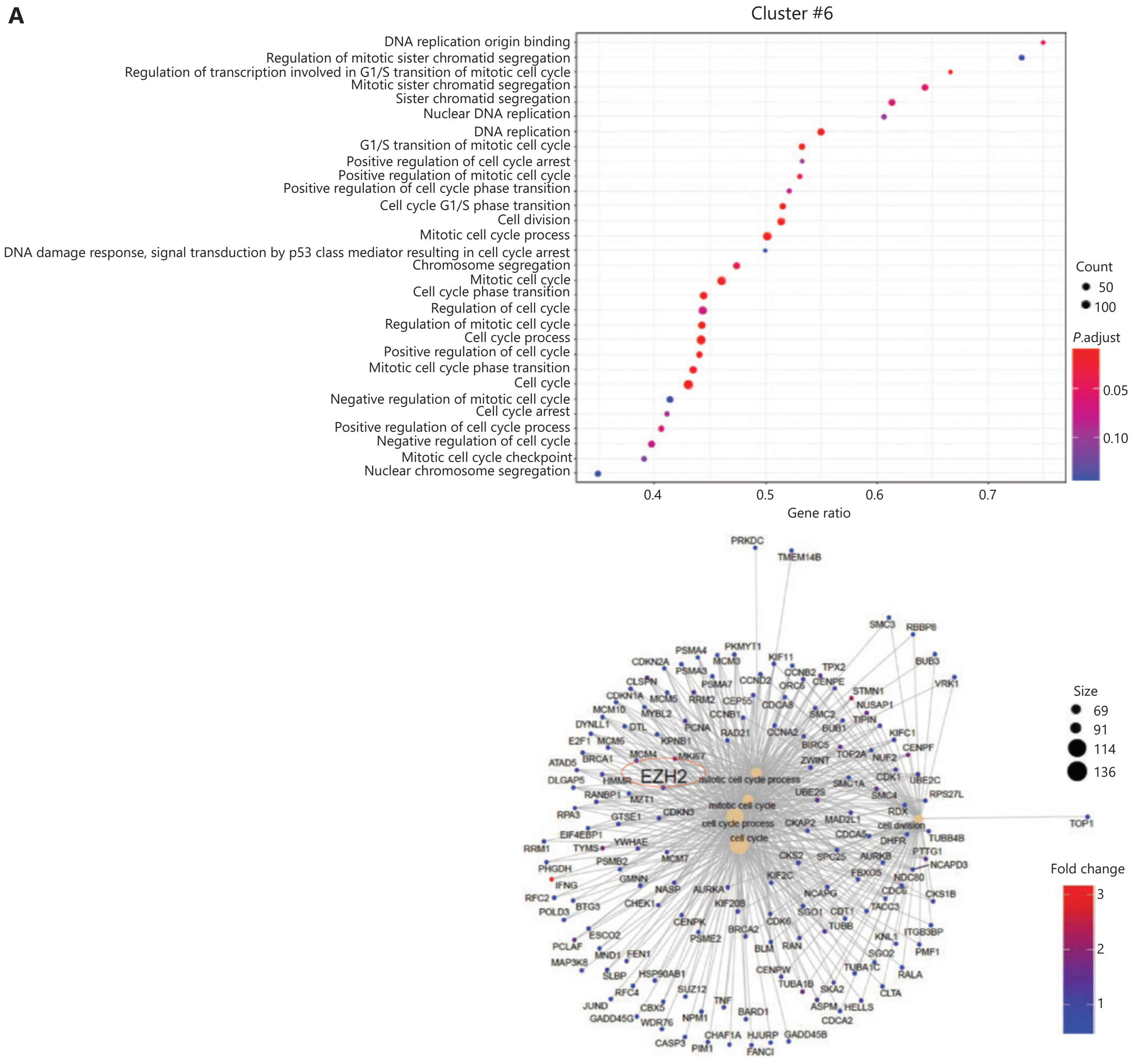

Figure S4 Continued

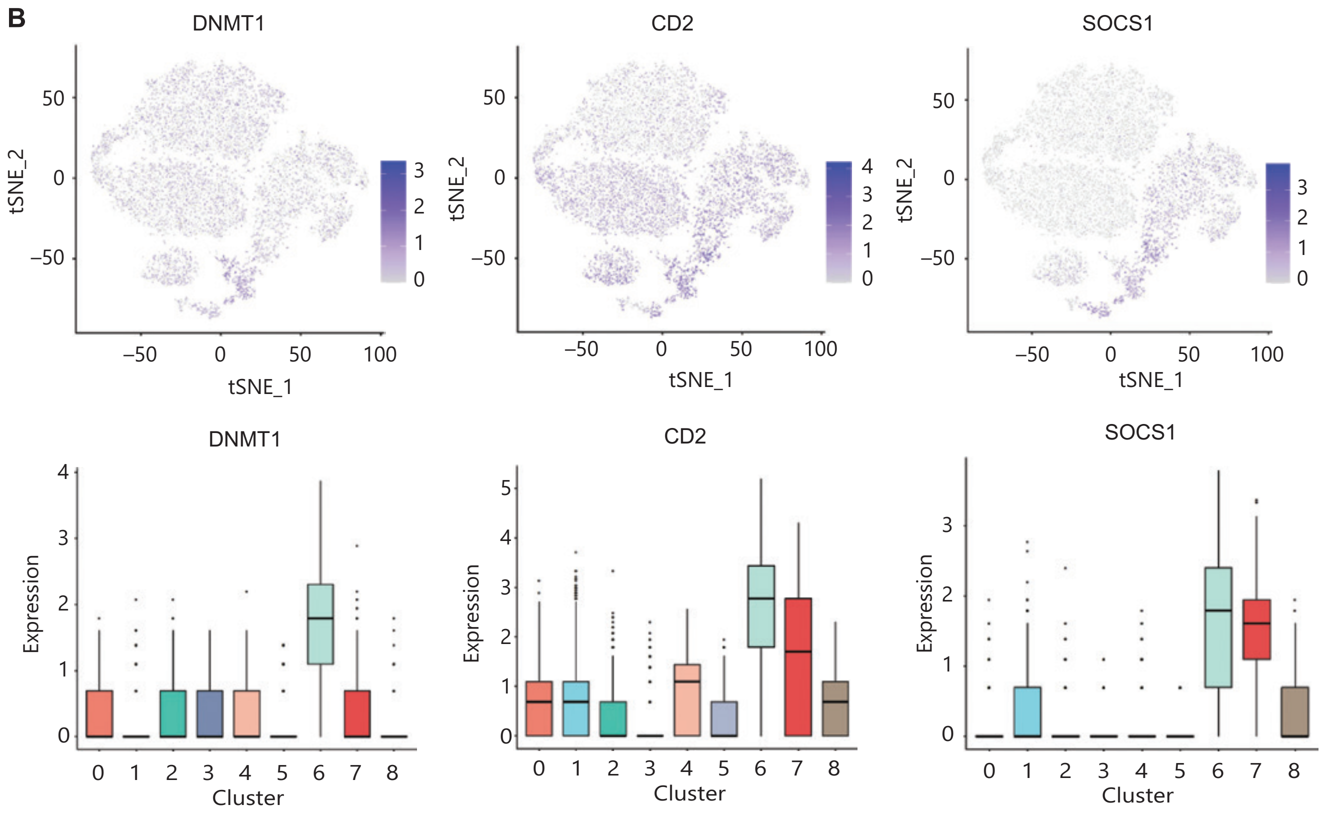

Figure S4 Characteristics of cluster #6 NK cells in IL12/18 pre-activated NK cells. (A) Cell cycle concentrated GOEA analysis of cluster #6 NK cells. Left, dot plot shows active pathways. Right, center plot shows active gene network. (B) *t*-SNE and box plots demonstrate the expression profile of DNMT1, CD2 and SOCS1 in all of the 9 NK clusters.

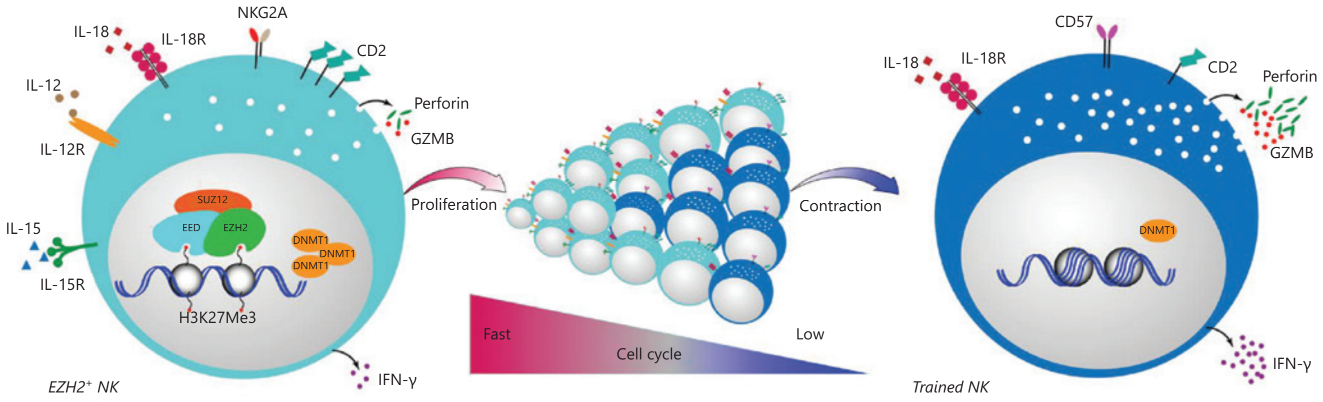

Figure S5 EZH2⁺ fast cell cycle NK precursor is the principal origin of cytokine induced trained NK. Our study of cytokine trained NK cells using scRNA-seq technology revealed a profile of cell surface markers and intracellular protein expression characteristic of the precursor of cytokine trained NK cells, and proposed a model for the differentiation of cytokine trained NK cells. These precursors we identified, intracellularly express PRC2 components EZH2, EED, SUZ12 and DNA methyltransferase DNMT1, which is responsible for its active proliferation ability and maintenance of its less differentiated DNA methylation status. On cell surface, these precursors express all cytokine receptors for IL-12, IL-15 and IL-18, inhibitory killer cell lectin-like receptor NKG2A and cell adhesion molecule CD2. The surface protein expression feature make these precursor sensitive to cytokine signal, restrained of killer function, and ready to accept co-stimulatory signal from antigen presenting cells *via* CD2-CD58 interaction. In the formation of cytokine trained NK cells, IL12/15/18 stimulation induce active proliferation of EZH2⁺ NK precursors, the proliferated progenies then differentiate into trained NK cells with enhanced function molecules expression (IFN-γ, GzmB and perforin), reduced proliferation ability (loss of PRC2 components and DNMT1 expression), reduced cytokine sensitivity (loss of IL-12R and IL-15R expression) and terminal differentiation marker expression (CD57).

Table S1 Donor information and sample information

Donor information			PBMC sample information		
Donor	Gender	Age	PBMC sample	Total NK viability (day 0)	Total NK viability (day 7)
Donor 1	Male	27	Human sample 1	98.4%	78.7%
Donor 2	Male	26	Human sample 2	97.7%	85.2%
Donor 3	Male	27	Human sample 3	97.9%	91.5%
